# DUSP2-mediated inhibition of tubular epithelial cell pyroptosis confers nephroprotection in acute kidney injury

**DOI:** 10.7150/thno.72291

**Published:** 2022-07-04

**Authors:** Jiachuan Xiong, Li Ran, Yingguo Zhu, Yaqin Wang, Shaobo Wang, Yue Wang, Qigang Lan, Wenhao Han, Yong Liu, Yinghui Huang, Ting He, Yan Li, Li Liu, Jinghong Zhao, Ke Yang

**Affiliations:** Department of Nephrology, the Key Laboratory for the Prevention and Treatment of Chronic Kidney Disease of Chongqing, Chongqing Clinical Research Center of Kidney and Urology Diseases, Xinqiao Hospital, Army Medical University (Third Military Medical University), Chongqing, 400037, P.R. China.

**Keywords:** Acute kidney injury, renal tubular epithelial cell, DUSP2, pyroptosis, STAT1

## Abstract

**Rationale:** Acute kidney injury (AKI) is pathologically characterized by renal tubular epithelial cell (RTEC) death and interstitial inflammation, while their pathogenesis remains incompletely understood. Dual-specificity phosphatase 2 (DUSP2) recently emerges as a crucial regulator of cell death and inflammation in a wide range of diseases, but its roles in renal pathophysiology are largely unknown.

**Methods:** The expression of DUSP2 in the kidney was characterized by histological analysis in renal tissues from patients and mice with AKI. The role and mechanism of DUSP2-mediated inhibition of tubular epithelial cell pyroptosis in AKI were evaluated both *in vivo* and *in vitro*, and confirmed in RTEC-specific deletion of DUSP2 mice.

**Results:** Here, we show that DUSP2 is enriched in RTECs in the renal tissue of both human and mouse and mainly positions in the nucleus. Further, we reveal that loss-of-DUSP2 in RTECs not only is a common feature of human and murine AKI but also positively contributes to AKI pathogenesis. Especially, RTEC-specific deletion of DUSP2 sensitizes mice to AKI by promoting RTEC pyroptosis and the resultant interstitial inflammation. Mechanistic studies show that gasdermin D (GSDMD), which mediates RTEC pyroptosis, is identified as a transcriptional target of activated STAT1 during AKI, whereas DUSP2 as a nuclear phosphatase deactivates STAT1 to restrict GSDMD-mediated RTEC pyroptosis. Importantly, DUSP2 overexpression in RTECs via adeno-associated virus-mediated gene transfer significantly ameliorates AKI.

**Conclusion:** Our findings demonstrate a hitherto unrecognized role of DUSP2-STAT1 axis in regulating RTEC pyroptosis in AKI, highlighting that DUSP2-STAT1 axis is an attractive therapeutic target for AKI.

## Introduction

Acute kidney injury (AKI) is a common and severe condition in which renal function declines rapidly in a short time 1. AKI is associated with high morbidity and mortality in hospitalized patients 2-5. Even if the patients survive, the post-AKI prognosis is not optimistic, with 30% to 70% developing chronic kidney disease and end-stage kidney disease 6, 7. Unfortunately, the effective therapeutic strategies for AKI remain limited due to the incomplete understanding of AKI pathogenesis 8.

Renal tubular epithelial cells (RTECs), which comprise the bulk of the renal parenchyma and position centrally in maintaining homeostasis through reabsorption, are particularly predisposed to ischemic and toxic insults 9. Sublethal and/or lethal damage of RTECs is a typical pathological characteristic of AKI 10. Of note, RTEC death is not merely a consequence of AKI, but is also identified as an essential pathogenic factor for AKI 11. Although the sublethal injury is reversible, the death of RTECs is inevitably accompanied by tubular dysfunction 12. More importantly, RTEC death frequently stimulates and even amplifies inflammation by releasing damage-associated molecular patterns and inflammatory cytokines, thereby further exacerbating RTEC injury and consequently promoting AKI progression 13. Therefore, targeting RTEC death is well recognized as a promising strategy to retard AKI progression. Although multiple modalities of RTEC death, such as apoptosis, ferroptosis, and pyroptosis, have been documented in AKI pathogenesis 14-16, the regulatory mechanisms of these distinctive cell death pathways during AKI remain largely unknown.

Dual-specificity phosphatase 2 (DUSP2), also known as phosphatase of activated cells 1 (PAC-1), inactivates protein kinases involved in cellular stress response, such as mitogen-activated protein kinases (MAPKs), through dephosphorylating dual threonine/tyrosine residues 17. Accordingly, DUSP2 plays essential roles in cell death and inflammation 18, 19. Previous studies have demonstrated that DUSP2 predominantly functions in activated immune effector cells, including T cells, B cells, macrophages, and mast cells. Interestingly, recent works reveal that DUSP2 is also actively implicated in the pathophysiological responses to toxins, hypoxia, and serum deprivation by several non-immune cells 20-22. However, with respect to renal cells, though the renal expression of DUSP2 was validated a decade ago 20, its functions in renal pathophysiology, including RTEC death and survival, are yet to be defined.

In the present study, we set out to characterize the role of DUSP2 in AKI pathogenesis. Using human biopsy samples and multiple mouse models of AKI, we reveal the preferential expression of DUSP2 in RTECs and that loss-of-DUSP2 in RTECs positively contributes to AKI pathogenesis. Functionally, DUSP2 restricts GSDMD-mediated RTEC pyroptosis through deactivating STAT1, thereby alleviating renal tubular injury and interstitial inflammation in AKI. Importantly, DUSP2 overexpression can significantly prevent AKI. Overall, these findings identify a hitherto unrecognized role of the DUSP2/STAT1 axis in regulating RTEC pyroptosis during AKI with promising translational potential.

## Methods

### Human samples

18 AKI patients who received renal biopsy from Xinqiao Hospital (Army Medical University, China) with various etiologies were enrolled in this study (Table S1). Paracarcinoma tissues from radical nephrectomy patients with no evidence of kidney injury through pathologic examination were used as a control. Written informed consent was acquired from all the patients. All procedures performed were in accordance with the ethical standards of the ethics committee of Xinqiao Hospital (IRB approval number 2018-006-01) and the principles of the Helsinki Declaration as revised in 2013. Renal biopsy sections from the AKI patients and paracarcinoma tissues were used for DUSP2 immunostaining staining. Then, the correlation of the expression of DUP2 with serum creatinine (Src), blood urine nitrogen (BUN), and renal injury biomarkers were analyzed.

### Animals

To generate tubule-specific DUSP2 knockout mice, *Dusp2^flox/flox^* (*Dusp2^fl/fl^*) mice were crossed with tubule-Cre mice (*Ggt1-cre* mice) to generate tubule-specific *Dusp2* knockout mice (*Ggt1-Cre^+^Dusp2^fl/fl^,* designated *Dusp2^CKO^*). *Dusp2^fl/fl^* littermates with no Cre expression served as controls. *Dusp2^CKO^* and* Dusp2^fl/fl^* male mice at around 8 weeks old were subjected to renal IR injury (IRI-AKI) or sham operation.

The IRI-AKI model was performed as described previously 23. In brief, the mice were anesthetized with 1% pentobarbital. After an abdominal incision was made, IRI was induced by clamping the renal pedicle of both kidneys for 35 min, and the clamps were then released. During the procedure, mice were warmed on a heating pad (36 °C) to maintain their body temperature. For sham operation, the kidneys were only exposed without clamped. For folic acid (FA) induced AKI model (FA-AKI model), male C57BL/6 mice intraperitoneally received 250 mg/kg FA or vehicle (0.3 M sodium bicarbonate) and were euthanized 2 days later. For cisplatin (CIS) induced AKI (CIS-AKI model), male C57BL/6 mice were intraperitoneally injected with 30 mg/kg cisplatin or vehicle (saline, 20 ml/kg) and were euthanized 3 days later. Given the commonality of decreased RTECs-expressive DUSP2 in distinct AKI models with different etiologies, we focused our subsequent mechanistic studies on IRI-AKI. Meanwhile, in terms of *Dusp2*/DUSP2 expressions over time after IR injury, day-2 after IR injury (IRI-D2) was selected. Blood samples were collected for further various analyses. All animal studies were performed according to the guiding principles established by the Institutional Animal Care and Use Committee (IACUC) of the Army Medical University.

### Cell culture

Human proximal tubular cells (HK-2 cells) were purchased from the American Tissue Culture Collection (ATCC, Manassas, VA, USA), and then cultured in DMEM/F12 (Dulbecco's Modified Eagle Medium with Ham's F12) medium supplemented with 10% fetal bovine serum (FBS, GibcoBRL, Rockville, MD, and 1% penicillin and streptomycin (Gibco, USA) under a humidified atmosphere consisting of 5% CO_2_ and 95% air at 37 °C.

### *Ex vivo* tubule culture

Mouse primary RTECs (pRTECs) culture was performed as previously described 24. Briefly, the cortical part of the kidneys from 4-week-old *Dusp2^fl/fl^* mice or AAV9-Dusp2 mice was collected, minced, and transferred to 10 mL HBSS (Hank's balanced salt solution). 0.1% collagenase II was added for 8 min, and then the digestion was terminated using the culture medium. Cells were filtered using a cell strainer (70 μm, Beyotime), and the suspension was centrifuged at 1000 rpm for 3min. These renal tubules were collected after an ultracentrifugation density gradient. After being washed with PBS, the precipitate was resuspended in DMEM/F12 (1:1) medium containing 10% FBS for 4-5 days.

### RNA interference

pRTECs from *Dusp2^fl/fl^* mice were transfected with HBAD-CRE-EGFP Cre-recombinase adenovirus (Ad-Cre-GFP) (Hanbio Inc, Shanghai, China). Briefly, pRTECs were isolated and cultured in 6-well plates for 4-5 days with a cell density of 60-70%. The medium was then replaced with 1 ml of serum-free medium in which recombinant adenovirus (MOI=50) was added to each well and incubated at 37 °C for 4 h. The medium was replenished and the virus-containing medium was aspirated after 6 h of infection and replaced with fresh complete medium, which was continued to be cultured at 37 °C. Infection efficiency was evaluated by observing the presence of GFP-positive cells under a fluorescence microscope.

pRTECs were separately transfected with Dusp2 plasmid (20 μM, GenePharma, Shanghai, China) or small interfering RNAs (siRNAs) (Table S2) including siSTAT1 (10 μM) or siGSDMD (10 μM, all Tsingke Biotechnology, Beijing, China) using Lipofectamine RNAiMAX Transfection Reagent (Invitrogen Life Technologies, Carlsbad, CA, USA) according to the manufacturer's instructions. The transfection efficiency was verified by qPCR.

The hypoxia/reoxygenation (H/R) was performed as follows. Before hypoxia (5% CO_2_ and 1% O_2_ at 37 °C), the medium was replaced with HBSS free medium (sugar-free and serum-free medium). Anoxic culture for 24 h, cells were transferred to 5% CO_2_ and 95% O_2_ for reoxygenation for another 6 h with a normal medium at 37 °C.

### Western blotting

Western blotting was performed as described previously 25. Briefly, the protein samples extracted from renal cortex, HK-2 cells and pRTECs were loaded onto sodium dodecyl sulfate-polyacrylamide gradient gels (SDS-PAGE) (Beyotime Biotechnology, Shanghai, China) and then transferred onto polyvinylidene fluoride (PVDF) membranes (Millipore, Billerica, MA, USA). The membranes were incubated with primary antibodies (listed in Table S3) at 4 °C overnight. The membrane was washed and incubated with goat anti-rabbit IgG (#7074; Cell Signaling, Danvers, MA, USA) or horse anti-mouse IgG antibody (#7076; Cell Signaling, Danvers, MA, USA) conjugated to horseradish peroxidase. The bands were analyzed using Image J software, normalized relative to β-actin, and the intensity for the phosphorylated proteins was normalized for the appropriate total protein.

### Quantitative real-time polymerase chain reaction (qPCR)

The sequences of primer pairs for different genes are shown in Table S4. qPCR was performed as described previously 25. Briefly, total RNA was isolated using the RNAeasy™ Plus Animal RNA Isolation Kit with Spin Column (R0032; Beyotime Biotechnology, Shanghai, China). The RNA was reverse transcribed into cDNA using the RT Master Mix for qPCR Kit (Cat. No: HY-K0511; MedChemExpress, Monmouth Junction, NJ). The target cDNA levels were quantified using qPCR, and mRNA levels were examined using a Bio-Rad CFX96 Detection System with SYBR Green PCR Master Mix (MedChemExpress, Monmouth Junction, NJ). The relative expression levels of each gene were normalized to the *β-Actin* gene.

### Immunohistochemistry and immunofluorescence staining

Paraffin-embedded mouse and human kidney sections (2.0-μm-thick) were prepared as described previously 25. A routine Hematoxylin-eosin (H&E) staining of the mouse kidney section was performed in accordance with protocol. DUSP2 expression was quantified using Image Pro-Plus v. 6.0 software (Bethesda, MD, USA).

Immunohistochemical (IHC) staining was performed as described previously 26. Briefly, after de-waxing, hydration, endogenous enzyme, and biotin removal and antigen repair, the sections were incubated with primary antibodies at 4 °C overnight. Then, the Cy3 (Goat Anti-Mouse IgG, Abcam; Goat anti-Rabbit IgG, Invitrogen)- or Alexa Fluor 488-conjugated-conjugated secondary antibody (Goat Anti-Mouse IgG, Abcam; Goat anti-Rabbit IgG, Invitrogen) were incubated for 1 h at room temperature before DAPI (Beyotime Biotechnology, Shanghai, China) staining. PBS was used as a negative control instead of a primary antibody. Slides were photographed using a confocal microscope (ZEISS, LSM780) and analyzed with ZEN 2012 software (version 1.1.1.0, Carl Zeiss Microscopy GmbH).

### Renal tubular injury score evaluation

The renal tubular injury score was evaluated as described previously 27. Briefly, two independent pathologists assessed the severity of renal tubule injury from 10 randomly selected fields from each renal tissue stained with hematoxylin and eosin. The renal tubular injury score was as follows, 0: normal; 1: mild injury, involvement of 0%-10%; 2: moderate injury, involvement of 11%-25%; 3: severe injury, involvement of 26%-49%; 4: high severe injury, involvement of 50%-75%; 5: extensive injury, involvement of >75%.

### Co-immunoprecipitation (Co-IP)

The Co-IP was performed according to the manufactural instruction (Pierce Classic Magnetic IP/Co-IP Kit, No, 88804, Thermo Fisher Scientific Inc.). Briefly, HK-2 cells treated with H/R were washed with pre-cooled PBS and lysed with ice-cold IP Lysis on ice for 5 min with periodic mixing. Then, Combine the cell lysate with anti-DUSP2, anti-STAT1, and anti-IgG (2 µg per 500 µg of total protein, A7028, Beyotime, China) antibodies, respectively. The cell lysate was incubated at room temperature for 1 hour to form the immune complex. Add the antigen sample/antibody mixture to the tube containing pre-washed magnetic beads and incubate at room temperature for 1 h with mixing after washing three times in 500 µL of IP lysis. Add 100 µL of elution buffer to the tube. Incubate the tube at room temperature with mixing for 10 min. Add 10 µL of neutralization buffer for each 100 µL of eluate to neutralize the low pH. The samples were separated on 10% SDS-PAGE for western blotting with anti-DUSP2 or anti-STAT1 antibody.

### Chromatin immunoprecipitation (ChIP)

ChIP was performed according to the manufacturer's instruction (Thermo Fisher Scientific, Santa Clara, CA, USA), as described previously 25. Briefly, HK-2 cells treated with H/R were incubated with 1% formaldehyde for crosslinking. Then, crosslinked HK-2 cells were incubated with a membrane extraction buffer containing a protease/phosphatase inhibitor. Cell lysis was then sonicated on ice with several pulses to break the nuclear membrane. The clipped cross-linked chromatin was co-precipitated with anti-DUSP2 antibody (Santa Cruz Biotechnology, CA) or IgG (as the controls) overnight. The harvested chromatin was then washed and incubated at 65 °C for 30 min with vigorous shaking. DNA Column was used to purify DNA, which was used for qPCR detection. The primers for ChIP are listed in Table S5.

### Transmission electron microscope and scanning electron microscopy

Samples were imaged respectively by a transmission electron microscope (TEM) (JEM-1400PLUS, Japan) and scanning electron microscopy (SEM) as previously described 28. Briefly, cells were fixed by 3% glutaraldehyde and 2% osmic acid successively. Then, cells were dehydrated by gradient ethanol and embedded with acetone. The paraffin-embedded cells were stained with uranium and captured using TEM. For SEM analyses, the cells were fixed with 2.5% glutaraldehyde overnight. The fixed cells were dehydrated, followed by hexamethyldisilane and immobilized onto a coverslip. The coverslip was sputtered with gold before observation at 15 kV under a Hitachi S-3400N SEM.

### Adeno-associated virus (AAV) 9 mouse model

The *Dusp2* (GenBank accession number NM_010090.2) cDNA was synthesized and purchased from GeneCopoeia (Rockville, Md, USA, Cat. no. AAV-Mm02227-AV08-A00) and an adeno-associated virus with serum type 9 (AAV9)-Dusp2 was also constructed and stored at -80 °C until use. qPCR was used to determine the number of physical copies of the viral genome. A total of 150 μl of sterile saline was injected into the tail vein of 6-week-old mice either with AAV9-Dusp2 or AAV9-Vector at 3×10^11^ genome copies/ml (GC/ml). The injected mice were fed and monitored for one month before they received IR treatment.

### Caspase-1/PI double staining

Pyroptosis was assessed using the Pyroptosis/caspase-1 double staining as previously described 29, and the Pyroptosis/caspase-1 assay (#9145, ImmunoChemistry Technologies, USA) was used according to the manufacturer's instructions. The pRTECs or HK-2 cells were collected and resuspended in FLICA solution after being washed twice with PBS. Cells were then incubated in the dark at room temperature for 1 h. After removing the wash buffer, the cells were resuspended in PI-containing reaction buffer for 15 min. Finally, flow cytometry was used to detect pyroptosis.

### Flow cytometric analysis

For detection of DUSP2 expression, the pRTECs treated with H/R were fixed using intracellular fixation buffer (eBioscience) and permeabilized using permeabilization buffer (eBioscience) before incubation with anti-human or mouse DUSP2 (Invitrogen, Carlsbad, CA, USA). For detection of IL-1β expression, single-cell suspension of the renal cortex was then prepared. After washing, the cells were resuspended in PBS and analyzed using a FACSverse (BD Biosciences, San Jose, CA, USA) flow cytometer. Data analysis was performed using FlowJo software (Treestar Inc, San Carlos, CA, USA).

### Scr and BUN measurements

Sham group and IRI group mouse blood samples were collected and centrifuged at 3000 rpm for 15 min. Then, upper serum samples were collected and analyzed. The levels of Scr and BUN were determined by using a commercial kit (C011-2; NJJCBIO, China) following the manufacturer's instructions as previously reported 26.

### Enzyme-linked immunosorbent assay

Cells were seeded into 24-well plates, and supernatants were collected before and after H/R treatment. Mouse IL-1β ELISA Kit (Boster Biological Technology, California, USA) was used according to the manufacturer's instructions. Sample and standard solution were added to each well and reacted for 90 min at 37 °C. Biotin-labeled antibody was added to each well and reacted for 60 min at 37 °C. In each well, 100 μl of ABC working solution was added, followed by 30 min at 37 °C. TMB substrate was therefore added at 37°C for 30 min under a dark environment. OD was measured using a microplate reader after adding TMB stop solution.

### Lactate dehydrogenase (LDH) release assay

LDH assay kit (MK401) was purchased from Takara Bio (Shiga, Japan). This experiment was performed in accordance with the instructions for the LDH cytotoxicity assay kit. Briefly, the supernatants of each treatment group were collected and centrifuged for 15 min. Afterward, 120 μl of supernatant from each well was added into a new 96-well plate, along with 60 μl of LDH detection working solution under dark conditions. The plate was then incubated at room temperature for 30 min, followed by LDH analysis using a multifunctional enzyme label analyzer at 490 nm.

### Statistical analysis

All values were expressed as mean±SD (standard deviation). A Student-t test was used to test the differences between 2 groups, while comparisons among multiple groups were performed with a one-way ANOVA followed by Tukey's post hoc test performed with GraphPad Prism software (version 9.3, San Diego, CA, USA). The correlation was calculated using Spearman's rank correlation coefficient. A p-value under 0.05 was considered significant. All the experiments were performed at least 3 times.

## Results

### Loss-of-DUSP2 in RTECs is common in AKI and positively contributes to AKI pathogenesis

**Figure 1:**
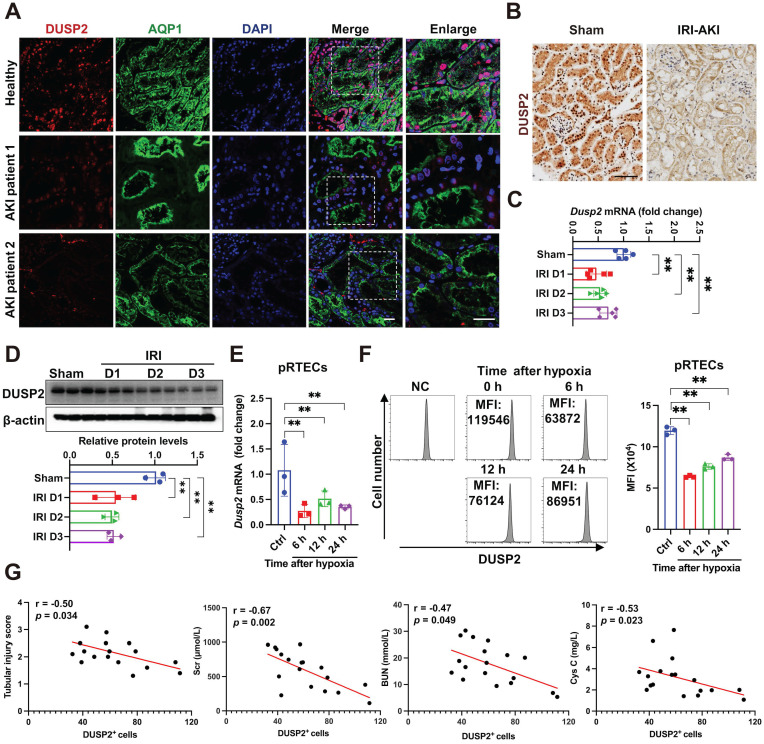
** Loss-of-DUSP2 in RTECs is common in AKI and positively contributes to AKI pathogenesis. (A)** Representative immunofluorescent images of DUSP2 in the kidney biopsies from patients with AKI. The paracarcinoma tissues from individuals without nephropathy were used as healthy controls. Scale bars, 20 µm. **(B)** Representative IHC staining of DUSP2 in the kidneys from sham and IRI-AKI mice. Scale bar, 50 µm. **(C-D)** Relative mRNA (C) (n = 5) and protein (D) expression levels of DUSP2 in renal cortex from sham and IRI mice (n = 3). **(E)** Relative mRNA expression levels of *Dusp2* in pRTECs after H/R injury (n = 3). **(F)** The expression levels of DUSP2 in pRTECs with or without H/R injury was evaluated by flow cytometry. **(G)** The correlation analysis between DUSP2 expression in RTECs and tubular injury score, Scr, BUN, or cystatin C in patients with AKI (n = 18). The average tubular DUSP2 positive cells were calculated based on 10 randomly selected fields from each kidney. Data are presented as mean±SD. *p < 0.05; **p < 0.01; compared with the indicated group.

Initially, using immunofluorescence, we observed that DUSP2 protein was preferentially expressed in RTECs from AKI patients, which are positively stained with AQP1, a marker of renal tubules (Figure 1A). To further identify the tubular segments that express DUSP2 in mice, double immunostaining for DUSP2 and segment-specific tubular markers was carried out and showed that DUSP2 is mainly colocalized with the proximal tubular marker (Figure S1A). Consistent with immune cells 18, DUSP2 was predominantly located in the nucleus of RTECs (Figure 1A), hinting that DUSP2 might be functional in RTECs. Interestingly, DUSP2 expression in RTECs was dramatically downregulated in patients with AKI (Figure 1A, Figure S1B). Similar results were also observed in several distinct mouse models of AKI, including IRI-AKI, cis-platinum-induced AKI (CIS-AKI), and folic acid-induced AKI (FA-AKI) (Figure 1B and Figure S1C-E), indicating that loss-of-DUSP2 in RTECs was a common characteristic of AKI. Moreover, loss-of-DUSP2 in RTECs occurred rapidly during AKI, being significant at 1 day post IRI and continuously declining till 3 days post IRI (Figure 1C-D). *In vitro*, DUSP2 expression was consistently reduced in either mouse pRTECs or human proximal tubular epithelial cell line HK-2 cells exposed to H/R, which is a widely-used *in vitro* model for IRI (Figure 1E-F, Figure S1F). More interestingly, in human AKI patients, the expression levels of DUSP2 in renal tubules were negatively correlated with renal injury markers, including renal tubular injury score, serum creatine (Scr), blood urea nitrogen (BUN), and cystatin C (Figure 1G), indicating that loss-of-DUSP2 in RTECs is closely associated with AKI pathogenesis. Collectively, these findings indicate that loss-of-DUSP2 in RTECs is common in AKI and positively contributes to AKI pathogenesis.

### RTEC-specific deletion of DUSP2 worsens AKI through sensitizing RTECs to cell death

**Figure 2:**
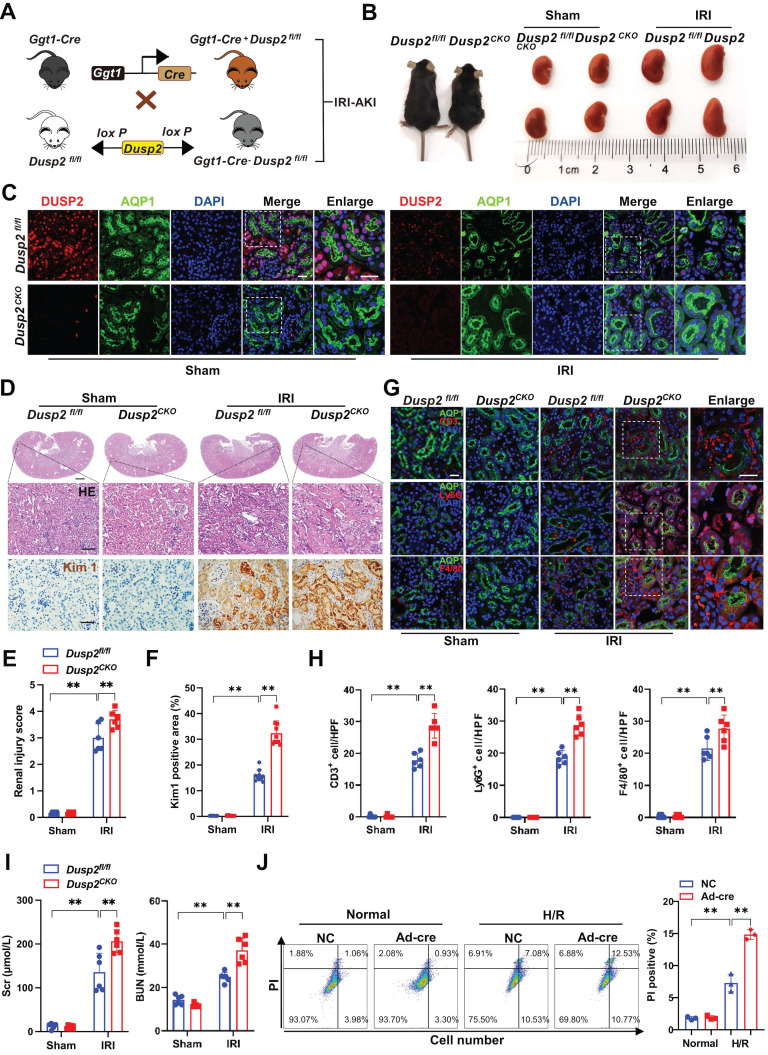
** RTEC-specific deletion of DUSP2 worsens AKI through sensitizing RTECs to cell death. (A)** Schematic diagram of *Dusp2^CKO^* mice generation before being subjected to IRI. **(B)** Renal morphology of *Dusp2^fl/fl^* and *Dusp2^CKO^* mice with or without IRI. **(C)** Representative immunofluorescent images of DUSP2 in the kidneys from *Dusp2^fl/fl^* and *Dusp2^CKO^* mice with or without IRI. Scale bars: 20 µm for low-power fields, 10 µm for high-power fields. **(D)** Representative images of H&E and IHC staining of Kim1 in the kidneys from *Dusp2^fl/fl^* and *Dusp2^CKO^* mice with or without IRI. Scale bars: 1 mm for renal morphology, 50 µm for H&E and IHC staining. **(E-F)** The tubular injury score (E) and quantitative analysis of Kim1 (F) in the kidneys from *Dusp2^fl/fl^* and *Dusp2^CKO^* mice with or without IRI (n = 6). **(G-H)** The expressions of CD3 (a marker for T cells), ly6G (a marker for neutrophils ), and F4/80 (a marker for macrophages) in the kidneys from *Dusp2^fl/fl^* and *Dusp2^CKO^* mice with or without IRI. Scale bar: 20 µm for low-power fields, 10 µm for high-power fields. **(I)** The measurements of Scr and BUN in *Dusp2^fl/fl^* and *Dusp2^CKO^* mice with or without IRI (n = 6). **(J)** The percentage of inflammatory cell death among pRTECs from *Dusp2^fl/fl^* mice infected with Ad-Cre-GFP prior to normoxia or H/R treatment. Data are presented as mean±SD. *p < 0.05; **p < 0.01; compared with the indicated group.

To define the role of DUSP2 in AKI pathogenesis, we generated RTEC-specific deletion of DUSP2 (*Dusp2^CKO^*) mice using a Cre-loxP recombination system involving *Ggt1-Cre* and* Dusp2^flox/flox^* (*Dusp2^fl/fl^*) mice (Figure 2A). RTEC-specific deletion of DUSP2 was confirmed by tail genotyping as well as qPCR and western blot analysis of mouse isolated RTECs (Figure S2A-C). Compared to their control littermates (*Dusp2^fl/fl^* mice), *Dusp2^CKO^* mice were born with normal renal morphology and functions (Figure 2B, Figure S2D). Subsequently, IRI was performed on *Dusp2^CKO^* (IRI/*Dusp2^CKO^*) mice and *Dusp2^fl/fl^* (IRI/*Dusp2^fl/fl^*) mice (Figure 2B). As shown, DUSP2 was hardly observed in RTECs of *Dusp2^CKO^* mice with or without IRI, although its expression was dramatically decreased in IRI/*Dusp2^fl/fl^* (Figure 2C). Of note, comparing to IRI/*Dusp2^fl/fl^* mice, the renal tubular injury was much more severe in IRI/*Dusp2^CKO^* mice, manifested by aggravated RTEC swelling and vacuolization, higher renal tubular injury score, and higher expression levels of renal tubular injury biomarkers, including kidney injury molecule 1 (*Kim1*) and neutrophil gelatinase-associated lipocalin (*Ngal*) (Figure 2D-F, Figure S2E). Besides, interstitial inflammation was also aggravated in IRI/*Dusp2^CKO^* mice, manifested by increased infiltration of inflammatory cells and overproduction of inflammatory cytokines (Figure 2G-H, Figure S2F). Consequently, renal dysfunction was worsened in IRI/*Dusp2^CKO^* mice, as reflected by higher serum levels of Scr and BUN (Figure 2I). These data indicate that RTEC-specific deletion of DUSP2 exacerbates renal tubular injury and the consequent interstitial inflammation. To exclude the developmental influence on the exacerbated tubular injury in *Dusp2^CKO^* mice post IRI, pRTECs from kidneys of *Dusp2^fl/fl^* mice were infected with Ad-Cre-GFP to specifically delete DUSP2 in RTECs before being subjected to H/R (Figure S2G-H). Surprisingly, we observed that pRTECs with loss-of-DUSP2 were vulnerable to cell death induced by H/R injury (Figure 2J). Given the central role of RTEC demise in driving AKI 30, these findings suggest that loss-of-DUSP2 exacerbates renal tubular injury and AKI progression probably through sensitizing RTECs to cell death.

### Loss-of-DUSP2 exacerbates RTEC pyroptosis by upregulating GSDMD during AKI

**Figure 3:**
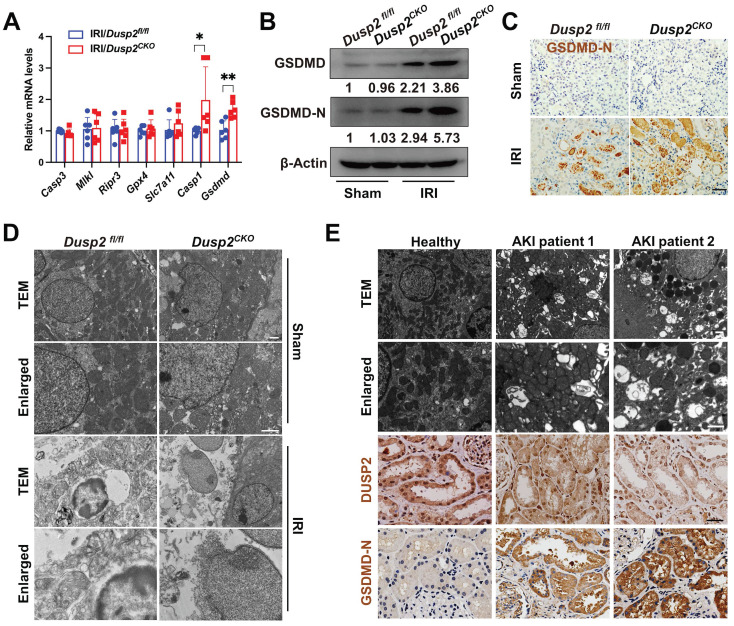
** Loss-of-DUSP2 exacerbates RTEC pyroptosis by upregulating GSDMD during AKI. (A)** Relative mRNA expression levels of the cell death executive genes in renal cortexes from *Dusp2^fl/fl^* and *Dusp2^CKO^* mice with IRI (n = 6). **(B)** The protein expression levels of GSDMD and GSDMD-N in renal cortexes from *Dusp2^fl/fl^* and *Dusp2^CKO^* mice with or without IRI. **(C)** Representative IHC staining of GSDMD-N in the renal cortexes from *Dusp2^fl/fl^* and *Dusp2^CKO^* mice with or without IRI. Scale bar, 50 µm. **(D)** Representative transmission electron microscopy (TEM) images of the kidneys from *Dusp2^fl/fl^* and *Dusp2^CKO^* mice with or without IRI. Scale bars: 1 µm for low-power fields, 0.5 µm for high-power fields. **(E)** Representative images of the TEM as well as IHC staining of DUSP2 and GSDMD-N in the kidney biopsies from healthy controls and patients with AKI. Scale bars: TEM, 1 µm for low-power fields, 0.5 µm for high-power fields. 50 µm for IHC staining.

RTECs will undergo multiple modalities of cell death during AKI. To determine the cell death pathway that was most affected by loss-of-DUSP2, we firstly extensively analyzed the executioners of apoptosis (*Caspase3*), necroptosis (*Mlkl* and *Ripr3*), ferroptosis (*Gpx4* and *Slc7a11*), and pyroptosis (*Casp1* and *Gsdmd*) in RTECs 31-35. Interestingly, comparing to IRI/*Dusp2^fl/fl^* mice, we found that only the executioner of pyroptosis (*Gsdmd*) was significantly upregulated in RTECs of IRI/*Dusp2^CKO^* mice (Figure 3A). Meanwhile, we also analyzed the expressions of *Gsdmd* and other gsdermin family members (*Gsdma*, *Gsdmc*, and *Gsdme*) *in vitro*, and found that DUSP2 deficiency significantly increased the mRNA levels of *Gsdmd* rather than *Gsdma*, *Gsdmc*, or *Gsdme*, in pRTECs post-H/R (Figure S3). GSDMD executes pyroptosis by the cleaved amino-terminal pore-forming domain of GSDMD (GSDMD-N) 34. Consistent with previous studies 36, we also detected significantly increased GSDMD protein and GSDMD-N in the renal tubules of IRI/*Dusp2^fl/fl^* mice (Figure 3B-C). Notably, the increases in GSDMD expression and activation were much more prominent in the renal tubules of IRI/*Dusp2^CKO^* mice (Figure 3B-C). Morphologically, pyroptosis is featured by membrane pores formation, cell swelling, and large bubbles blowing from the plasma membrane 37. Correspondingly, these characteristics were more evident in IRI/*Dusp2^CKO^* mice (Figure 3D). Besides, pyroptosis was also significantly induced in the renal tubules of AKI patients, and the severity of pyroptosis was negatively associated with DUSP2 expression in the renal tubules (Figure 3E).

**Figure 4:**
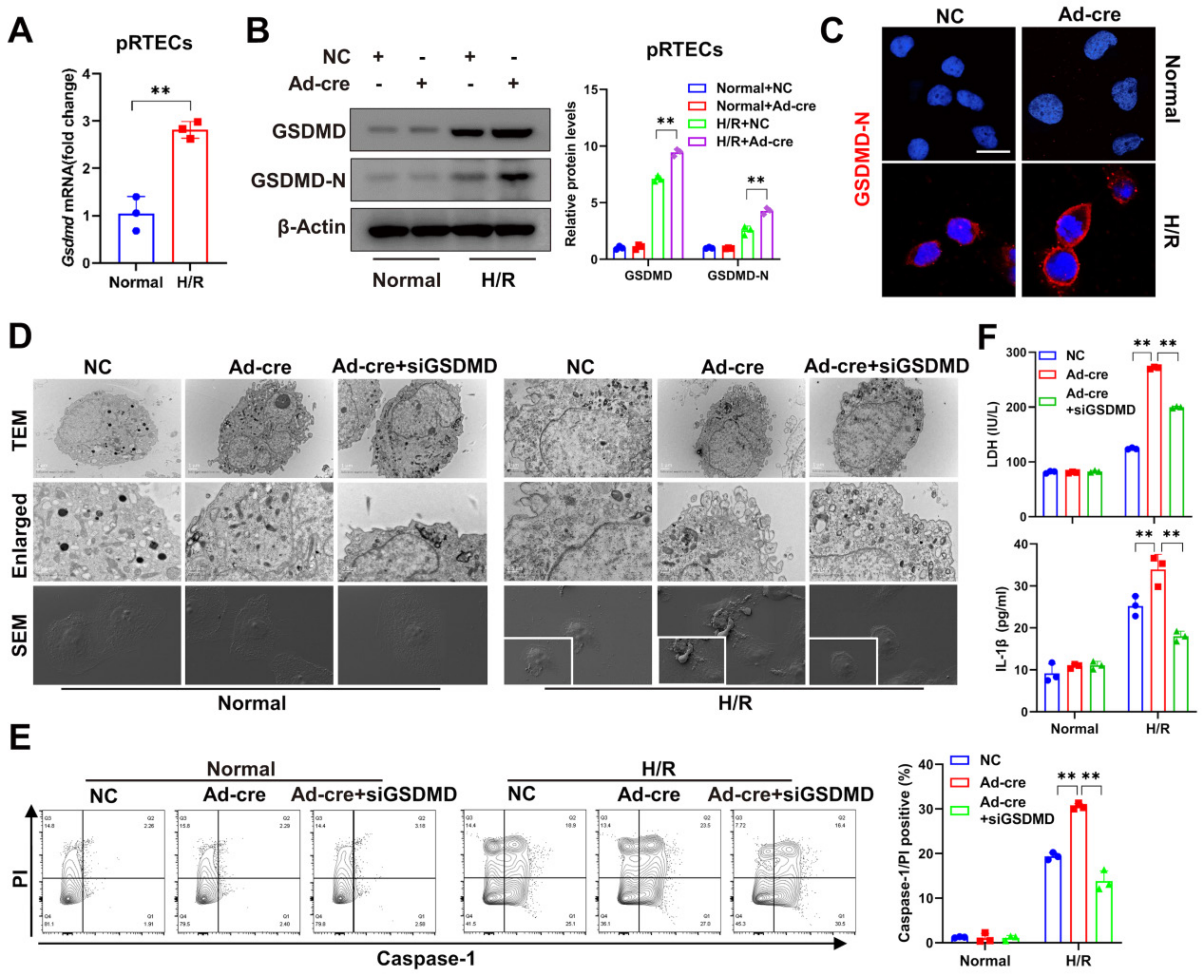
** DUSP2-deficient pRTECs increase pyroptosis post H/R. (A)** Relative mRNA expression levels of *Gsdmd* in pRTECs with or without H/R injury. **(B)** The protein expression levels of GSDMD and GSDMD-N in DUSP2-deficient pRTECs with or without H/R injury. **(C)** Representative immunofluorescent images of GSDMD-N in DUSP2-deficient pRTECs with or without H/R injury. Scale bars, 20 µm. **(D)** Representative TEM and SEM images of pyroptosis in DUSP2-deficient pRTECs with or without GSDMD RNAi prior to normoxia or H/R treatments. Scale bar: TEM, 1 µm for low-power fields, 0.5 µm for high-power fields. Magnification for SEM: ×500 for low-power fields, ×1000 for high-power fields. **(E)** The percentage of pyroptotic cell death among DUSP2-deficient pRTECs with or without GSDMD RNAi prior to normoxia or H/R treatments. The pyroptotic cell death was represented by the percentage of PI and caspase-1 double-positive cells. **(F)** The measurements of LDH and IL-1β in DUSP2-deficient pRTECs with or without GSDMD RNAi prior to normoxia or H/R treatments. Data are presented as mean±SD. *p < 0.05; **p < 0.01; compared with the indicated group.

*In vitro*, significantly increased pyroptosis was also observed in DUSP2-deficient pRTECs post-H/R (Figure 4A-E). Pyroptosis, which culminates in lytic cell death, also accompanies with the release of LDH and inflammatory cytokines such as IL1β 38. Indeed, we also detected markedly increased LDH and IL1β in the supernatant of DUSP2-deficient pRTECs (Figure 4F). Consistent with the *in vivo* results, loss-of-DUSP2 significantly upregulated GSDMD expression in RTECs, accompanied by increased activation of GSDMD, which eventually sensitized RTECs to pyroptosis induced by H/R *in vitro*. However, knockdown of GSDMD by RNA interference (RNAi) dramatically alleviated the enhanced sensitivity of RTECs with loss-of-DUSP2 to H/R-induced pyroptosis (Figure 4D-F). Altogether, these data demonstrate that loss-of-DUSP2 exacerbates RTEC pyroptosis through upregulating GSDMD expression. Given that pyroptosis as a form of inflammatory cell death can release both inflammatory cytokines such as IL1β and DAMPs to amplify inflammation 39, which is a key aggravating factor of AKI 40. These findings indicate that loss-of-DUSP2 exacerbates renal tubular injury and AKI progression through sensitizing RTECs to pyroptosis.

### STAT1 transactivates GSDMD to promote RTEC pyroptosis during AKI

**Figure 5:**
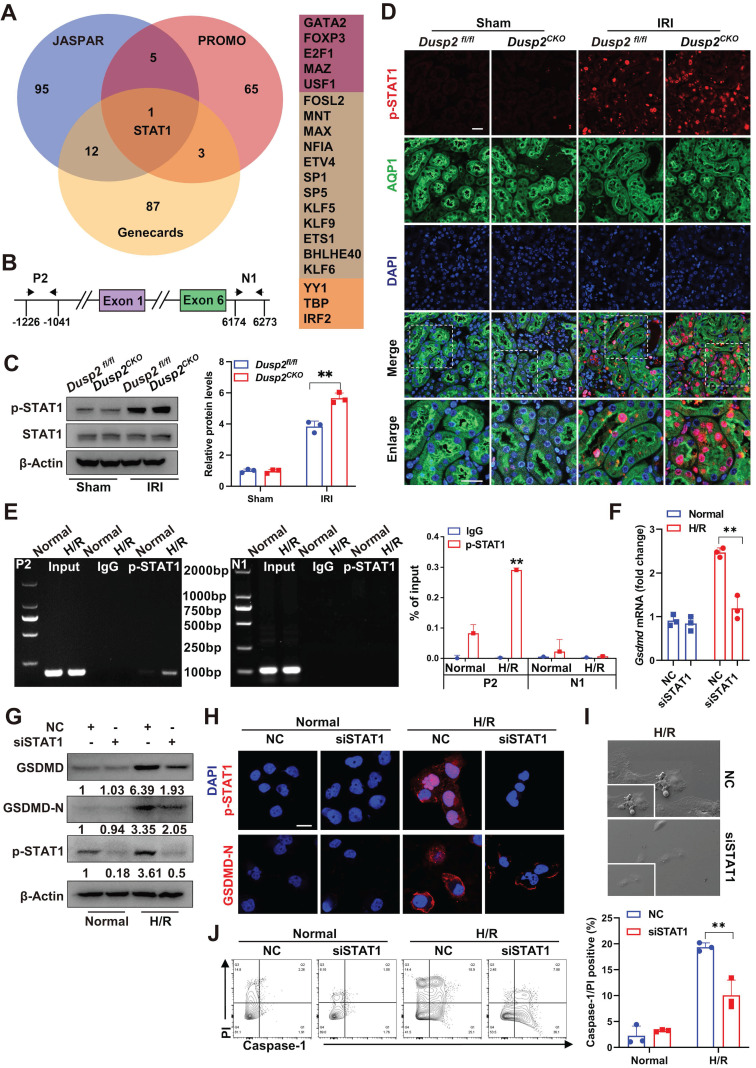
** STAT1 transactivates GSDMD to promote RTEC pyroptosis during AKI. (A)** The bioinformatics analysis (JASPAR, PROMO, and Genecards databases) of the predicted transcription factors bounding to *Gsdmd* promoter regions. **(B)** The promoter and intron regions (primer P2 and N1 were selected for the experiment) of *Gsdmd*. ChIP-PCR primer locations were marked using black arrows. The transcriptional start site was considered to be +1 in the promoter region. **(C)** The protein expression levels of STAT1 and p-STAT1 in the renal cortexes from *Dusp2^fl/fl^* and *Dusp2^CKO^* mice with or without IRI. **(D)** Representative immunofluorescent images of p-STAT1 in the kidneys from *Dusp2^fl/fl^* and *Dusp2^CKO^* mice with or without IRI. Scale bars: 20 µm for low-power fields, 10 µm for high-power fields. **(E)** The ChIP assay between GSDMD and human p-STAT1. Data were expressed as the percentage of input DNA. **(F)** The mRNA expression of *Gsdmd* in pRTECs treated with or without STAT1 RNAi prior to normoxia or H/R treatments. **(G)** The protein expression levels of GSDMD-N, GSDMD, and p-STAT1 in the pRTECs treated with or without STAT1 RNAi prior to normoxia or H/R treatments. **(H)** Representative immunofluorescent images of p-STAT1 and GSDMD-N in the pRTECs with or without STAT1 RNAi prior to normoxia or H/R treatments. Scale bar, 20 µm. **(I)** Representative SEM images of pyroptosis in the pRTECs treated with or without STAT1 RNAi before H/R injury. Magnifyication for SEM: ×500 for low-power fields, ×1000 for high-power fields. **(J)** The percentage of pyroptotic cell death among pRTECs with or without STAT1 RNAi prior to normoxia or H/R treatments. Data are presented as mean±SD. *p < 0.05; **p < 0.01; compared with the indicated group.

Interestingly, the nuclear localization of DUSP2 in RTECs excluded the direct regulation of cytosolic GSDMD, hinting that DUSP2 may modulate GSDMD expression by influencing its transcription factors' activity. Therefore, we performed bioinformatical analysis to predict the potential transcription factors of *Gsdmd* based on three distinct databases, including PROMO, Genecards, and JASPAR. Intriguingly, all the three databases collectively predicted that the signal transducer and activator of transcription 1 (STAT1) might be the most promising transcription factor for *Gsdmd* (Figure 5A). Besides, a potential binding site of STAT1 on the promotor region of* Gsdmd* was also found (Figure 5B).

As STATs (STAT1, STAT3, and STAT5) play an important role in regulating cell death, we thus detected the alterations of STATs in *Dusp2^CKO^* mice with or without IRI. The expression and phosphorylation of STAT1, but not those of STAT3 and STAT5, were observed in kidneys from IRI/*Dusp2^CKO^* mice (Figure 5C-D, Figure S4A), further defining that DUSP2 deficiency in RTECs primarily contributes to the activation of STAT1 protein in kidneys post-IRI. As known, STAT1 protein is conventionally phosphorylated by tyrosine or serine residues, such as tyrosine 701 (Tyr^701^), serine 726 (Ser^726^), and Ser^727^. Among these residues, phosphorylating at Tyr^701^ or Ser^727^ sites allow the phosphorylated STAT1 to translocate into the nucleus, where it binds to promoter regions and influences the gene transcription 41-42. Consequently, we created two STAT1 mutants with substitution of Tyr^701^ and Ser^727^. These two mutant plasmids of STAT1 were subjected to site-directed mutagenesis in HK-2 cells post-H/R with or without DUSP2 overexpression and the phosphorylation of STAT1 was evaluated. The defect of STAT1 phosphorylation was more pronounced at Tyr^701^ mutation than those at Ser^727^ mutation post-H/R, indicating that H/R primarily indudced Tyr^701^-phosphorylation of STAT1 (Figure S4B).

Using ChIP, we observed that p-STAT1 could directly bind to the *Gsdmd* promoter, and the binding was significantly enhanced post H/R (Figure 5E). On the other hand, knockdown of STAT1 significantly abolished the upregulation and activation of GSDMD (Figure 5F-H, Figure S4C), and finally alleviated RTEC pyroptosis induced by H/R (Figure 5I-J). These results indicate that STAT1-mediated GSDMD transactivation substantially contributes to RTEC pyroptosis during AKI.

### DUSP2 deactivates STAT1 to restrict GSDMD-mediated RTEC pyroptosis

**Figure 6:**
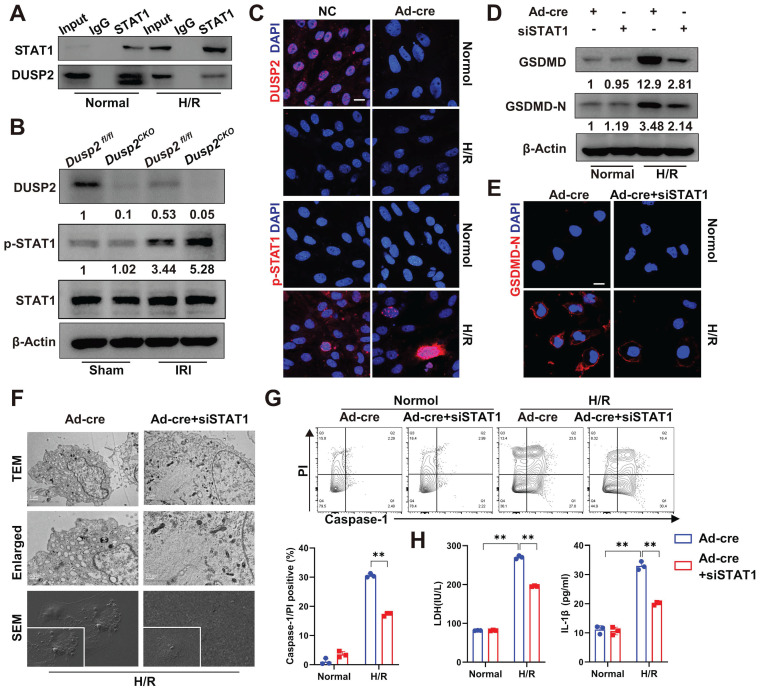
** DUSP2 deactivates STAT1 to restrict GSDMD-mediated RTEC pyroptosis. (A)** Co-IP assays between DUSP2 and STAT1 in RTECs with or without H/R injury. **(B)** The protein expression levels of DUSP2, p-STAT1, and STAT1 in renal cortexes from *Dusp2^fl/fl^* and *Dusp2^CKO^* mice with or without IRI. **(C)** Representative immunofluorescent images of DUSP2 and p-STAT1 in the DUSP2-deficient pRTECs with or without H/R injury. Scale bar, 20 µm. **(D-E)** The expression levels of GSDMD and GSDMD-N in DUSP2-deficient pRTECs with or without STAT1 RNAi prior to normoxia or H/R treatments. Scale bars, 20 µm. **(F)** Representative TEM and SEM images of pyroptosis in the DUSP2-deficient pRTECs with or without STAT1 RNAi before H/R injury. Scale bar: TEM, 1 µm for low-power fields, 0.5 µm for high-power fields. Magnification for SEM: ×500 for low-power fields, ×1000 for high-power fields. **(G)** The percentage of pyroptotic cell death in DUSP2-deficient pRTECs with or without STAT1 RNAi prior to normoxia or H/R treatments. **(H)** The measurements of LDH and IL-1β in the DUSP2-deficient pRTECs with or without STAT1 RNAi prior to normoxia or H/R treatments. Data are presented as mean±SD. *p < 0.05; **p < 0.01; compared with the indicated group.

Next, we hypothesized that DUSP2 as a nuclear phosphatase might negatively regulate GSDMD expression by combining physically with and dephosphorylating STAT1. Indeed, Co-IP analysis showed that FLAG-tagged DUSP2 could be precipitated by endogenous STAT1 in RTECs (Figure 6A), confirming the physical association between DUSP2 and STAT1. Notably, the dephosphorylation of DUSP2 on Tyr^701^-phosphorylation of STAT1 was more evident than its effect on Ser^727^-phosphorylation after H/R injury, demonstrating that DUSP2-mediated dephosphorylation of STAT1 probably mainly occurs at Tyr^701^ site (Figure S4B). However, the physical association between DUSP2 and STAT1 was weakened in RTECs post-H/R, accompanied by dramatic phosphorylation of STAT1 (Figure 6A-C). Moreover, STAT1 phosphorylation was further enhanced in RTECs with DUSP2 deficiency both *in vivo* and *in vitro* (Figure 6B-C, Figure S4D). Conversely, knockdown of STAT1 significantly abrogated the upregulation and activation of GSDMD in RTECs with DUSP2 deficiency (Figure 6D-E), and finally attenuated the enhanced pyroptosis in RTECs with DUSP2 deficiency post H/R (Figure 6F-H). Taken together, these results demonstrate that DUSP2 as a nuclear phosphatase deactivates STAT1 to restrict GSDMD-mediated RTEC pyroptosis.

### DUSP2 overexpression in RTECs protects against AKI

**Figure 7:**
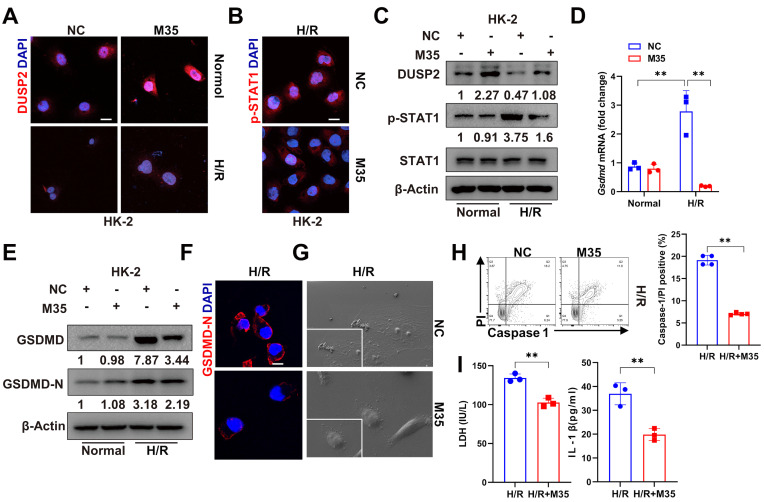
** DUSP2 overexpression counteracted H/R-induced activation of STAT1 and GSDMD in RTECs. (A-B)** Representative immunofluorescent images of DUSP2 (A) and p-STAT1 (B) in HK-2 cells with or without DUSP2 overexpression prior to normoxia or H/R treatments. Scale bar, 20 µm. **(C)** The protein expression levels of DUSP2, p-STAT1, and STAT1 in HK-2 cells with or without DUSP2 overexpression prior to normoxia or H/R treatments. **(D)** The mRNA expression of *Gsdmd* in HK-2 cells with or without DUSP2 overexpression prior to normoxia or H/R treatments. **(E-F)** The expression levels of GSDMD and GSDMD-N in HK-2 cells with or without DUSP2 overexpression prior to normoxia or H/R treatments. Scale bars, 20 µm. **(G)** Representative SEM images of the HK2 cells with or without DUSP2 overexpression before H/R injury. Magnification for SEM: ×500 for low-power fields, ×1000 for high-power fields. **(H-I)** The percentage of pyroptotic cell death (H) as well as the measurements of LDH and IL-1β (I) in HK2 cells with or without DUSP2 overexpression before H/R injury. Data are presented as mean±SD. *p < 0.05; **p < 0.01; compared with the indicated group.

**Figure 8:**
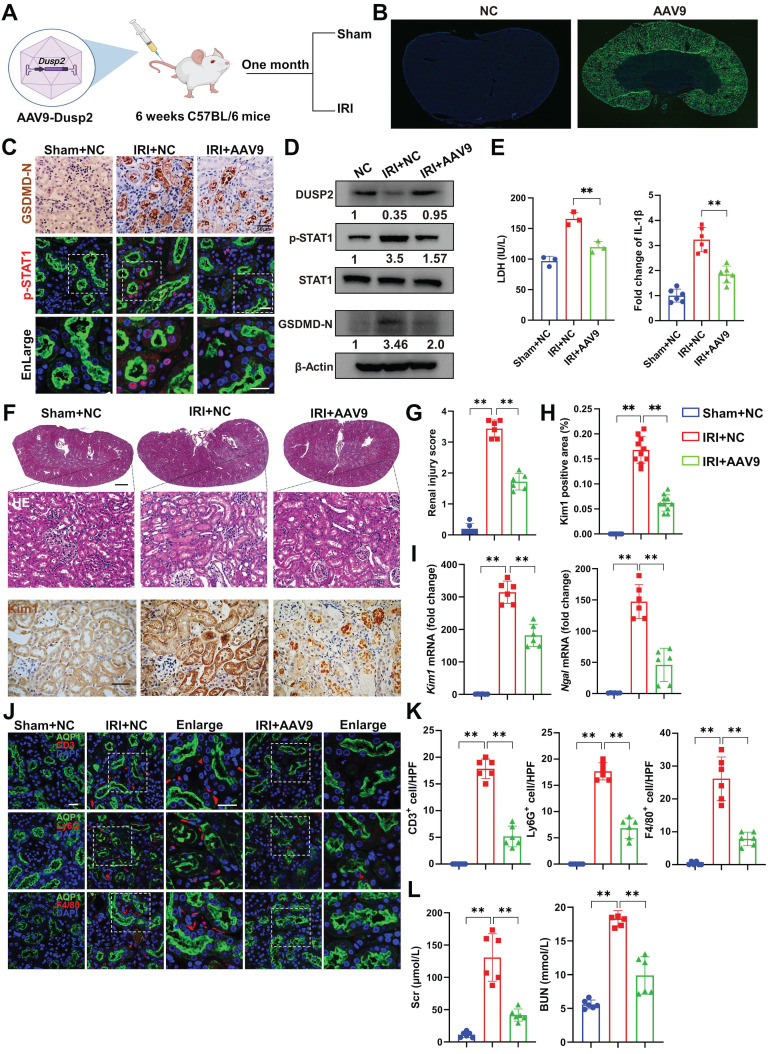
** Overexpression of DUSP2 mitigates RTECs injury in AKI. (A)** Schematic diagram of the recombinant adeno-associated vector type 9 expressing *Dusp2* (AAV9-*Dusp2*) administration in C57BL/6J mice for one month before being subjected to IRI. **(B)** Representative immunofluorescent images of the kidneys from mice injected with or without AAV9-*Dusp2*. **(C-D)** The expression levels of GSDMD-N, p-STAT1, STAT1, and DUSP2 in the renal cortexes from mice injected with or without AAV9-*Dusp2* before being subjected to IRI. **(E)** The measurements of LDH and IL-1β in the renal cortex from mice injected with or without AAV9-*Dusp2* before being subjected to IRI. **(F)** Representative images of H&E and IHC staining of Kim1 in the kidneys from mice injected with or without AAV9-*Dusp2* before being subjected to IRI. Scale bars: 1 mm for renal morphology, 50 µm for H&E and IHC staining. **(G-H)** The renal injury scores (G) and quantitative analysis of Kim1 (H) in the kidneys from mice injected with or without AAV9-*Dusp2* before being subjected to IRI (n = 6). **(I)** Relative mRNA expression levels of *Kim1* and *Ngal* in the renal cortexes from mice injected with or without AAV9-*Dusp2* before being subjected to IRI (n = 6). **(J-K)** The expressions of CD3, ly6G, and F4/80 in the kidneys from mice injected with or without AAV9-*Dusp2* before being subjected to IRI (n = 6). Scale bars, 20 µm. **(L)** The measurements of Scr and BUN in the mice injected with or without AAV9-*Dusp2* before being subjected to IRI (n = 6). Data are presented as mean±SD. *p < 0.05; **p < 0.01; compared with the indicated group.

Finally, we interrogated whether DUSP2 overexpression in RTECs could prevent RTEC pyroptosis and consequently ameliorate AKI. We firstly overexpressed DUSP2 in HK-2 cell by M35 (*Dusp2* plasmid) (Figure 7A-B, Figure S5A). It was found that DUSP2 overexpression significantly counteracted H/R-induced activation of STAT1 as well as upregulation and activation of GSDMD in RTECs, and finally alleviated pyroptosis post H/R (Figure 7B-I). Furthermore, to evaluate the translational potential of our findings, mice with DUSP2 overexpressed that was achieved by adeno-associated virus-mediated gene transfer (AAV9-*Dusp2*) were subjected to IRI (Figure 8A). Consistently, we observed that the increased DUSP2 was mostly enriched in the renal cortex, and was much higher in renal tubules from the AAV9-Dusp2-transfected mice than those from NC mice (Figure 8B, Figure S5B-D). As did the results *in vitro*, the activation of STAT1, as well as the upregulation and activation of GSDMD, were significantly restrained in the renal tubules of AAV9-*Dusp2* mice, accompanied by mitigated RTEC pyroptosis (Figure 8C-E, Figure S5E). Consequently, the renal tubules of AAV9-*Dusp2* mice exhibited significantly alleviated morphology of injuries such as swelling and vacuolation, together with lower levels of *Kim1* and *Ngal* (Figure 8F-I). Meanwhile, the interstitial inflammation was also remarkably mitigated in AAV9-*Dusp2* mice post IRI (Figure 8J-K, Figure S5F). As a result, the renal function was significantly preserved in AAV9-*Dusp2* mice post IRI (Figure 8L). Furthermore, we also explored the renoprotective role of DUSP2 post-AKI. After AAV9-Dusp2 transfection, the renal injury and interstitial fibrosis were observed in day-14 after subjecting to IRI injury. In contrast to the NC mice, the renal injury and inflammation as well as interstitial fibrosis were dramatically alleviated after AAV9-Dusp2 treatment, suggesting that DUSP2 could protect kidney against renal fibrosis post-AKI (Figure S6). Thus, these data demonstrate that restoration of DUSP2 expression in RTECs can protect against AKI and may represent a promising therapeutic strategy.

## Discussion

**Figure 9:**
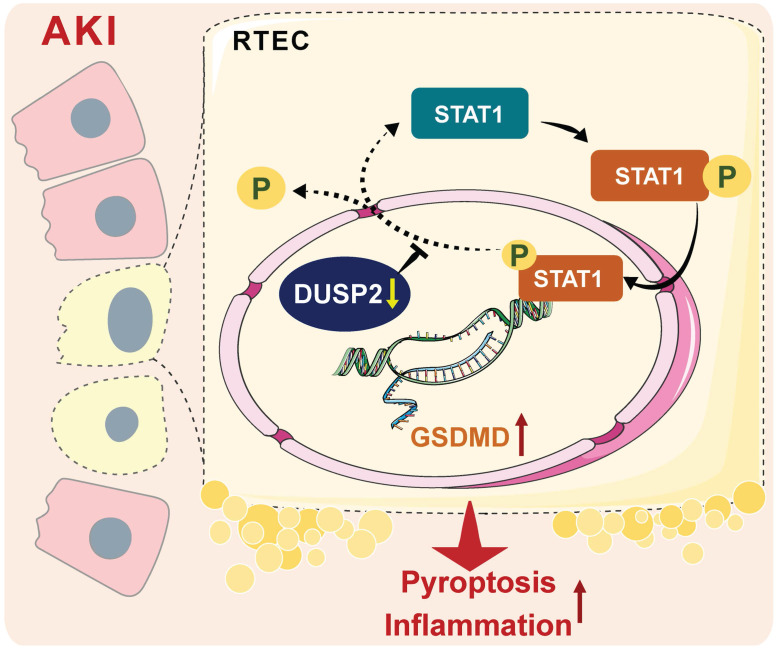
A schematic illustrating the proposed function of DUSP2 in the regulation of pyroptosis. During AKI insults, the renal tubular DUSP2 expression is reduced, hampers the dephosphorylating of STAT1. Then the phosphorylated STAT1 can directly promote GSDMD transcription and expedite pyroptosis and subsequent inflammation.

RTEC death and inflammation are well-known characteristics of AKI, while their pathogenesis is incompletely understood. In the present study, loss-of-DUSP2 in RTECs is identified as a common characteristic and a prognostic factor of human and murine AKI. Functionally, DUSP2 is identified as a deactivator of STAT1 in RTECs, whose deficiency permits STAT1 hyperactivation and thereby promotes RTEC pyroptosis through transactivating GSDMD during AKI. Therapeutically, DUSP2 overexpression effectively protects against AKI (Figure 9). Overall, our study substantially extends our understanding of AKI pathogenesis and provides promising therapeutic targets for AKI.

DUSPs, which consist of 25 family members, are well known to be implicated in the pathogenesis and/or development of many diseases, including kidney diseases 43. With respect to DUSP2, which actively regulates the immune-inflammation process 44, a key episode of AKI 45, its pathophysiological role in AKI remains scarcely recognized. Here, we show that DUSP2 is primarily enriched in RTECs among the renal inherent cells and mainly positions in the nucleus. Using multiple murine AKI models, together with renal biopsy samples from AKI patients, we reveal that DUSP2 expression is dramatically downregulated in RTECs during AKI and that loss-of-DUSP2 may be a common feature of AKI. Further, we disclose a positive correlation between DUSP2 expression in renal tubules and renal function within AKI patients, hinting a pivotal role of DUSP2 in the pathogenesis and development of AKI. Indeed, when DUSP2 is specifically deleted in RTECs, though mice are born with a normal phenotype, renal dysfunction is significantly aggravated in IRI-induced AKI, accompanied by much more severe renal tubular injury and interstitial inflammation. Therefore, DUSP2 acts as a key governor of RTEC fitness during AKI. As reported, hypoxia, which is a primary pathogenic factor of AKI and our AKI models 46, is a strong inducer of DUSP2 downregulation through the activation of hypoxia-inducible factor (HIF) 47-49. Since HIF is also activated in RTECs during AKI 50, this may explain the downregulation of DUSP2 in RTECs during AKI.

Pyroptosis is a regulated cell death pathway that results in the profound activation of inflammatory cascade due to membrane rupture 51. Emerging evidence shows that pyroptosis is also implicated in the pathogenesis of AKI and contributes substantially to renal injury and inflammation 52. In particular, RTEC pyroptosis has been identified as a key episode during IRI, targeting which can protect against AKI that is induced by various insults 53-55. Unfortunately, although the execution of pyroptosis is well established in RTECs during AKI, its regulatory mechanism remains poorly understood. In this study, we uncover that loss-of-DUSP2 distinctively sensitizes RTECs to pyroptosis during IRI-induced AKI, highlighting a regulatory role of DUSP2 in RTEC pyroptosis during AKI. Causally, this unrecognized action of DUSP2 explains the much more severe RTEC injury and interstitial inflammation in the murine AKI model with RTEC-specific deletion of DUSP2. On the contrary, AAV9-mediated DUSP2 overexpression in RTECs potently prevents RTEC pyroptosis and interstitial inflammation, and finally protects against IRI-induced AKI. Thus, our study strongly suggests that DUSP2 can be developed as a therapeutic target for AKI.

Gasdermins, including GSDMA, GSDMB, GSDMC, GSDMD, and GSDME, are the executors of pyroptosis 56. Consistent with the previous studies 36, 57, we confirm that GSDMD mainly mediates RTEC pyroptosis during IRI-induced AKI. Surprisingly, loss-of-DUSP2 further increases GSDMD expression and activation in RTECs during IRI-induced AKI, suggesting that GSDMD may act downstream of DUSP2. However, the nuclear localization of DUSP2 in RTECs excludes the direct regulation of cytosolic GSDMD. We assume that an indirect regulation of GSDMD by DUSP2 maybe exist. Using bioinformatics screening, our study for the first time identifies that GSDMD is a transcriptional target of STAT1. On the other hand, DUSP2 is well known as a phosphatase that deactivates a wide range of phosphorylated transcription factors such as MAPKs, focal adhesion kinase (FAK), lymphocyte-specific protein tyrosine kinase (LCK), and STAT3 17, 58, 59. Interestingly, in the present study, we further show that DUSP2 can physically associate with and dephosphorylate activated STAT1 at the Tyr^701^-phosphorylation site in RTECs. Of note, the reported substrates of DUSP2, such as Erk1/2 or p38 MAPK and STAT3, have also been found to be phosphorylated in the kidney during AKI 60-62. However, DUSP2 always forms homodimers or forms complexes with other DUSPs, thereby giving rise to different substrate preferences. For example, the DUSP2-DUSP4 heterodimer is more closely related to STAT3 than did the DUSP2-DUSP2 homodimer 58. Additionally, DUSP2 preferentially regulates different signaling pathways in different milieus 17, indicating that different stimuli affect the substrate specificity of DUSP2. It is thus conceivable that DUSP2 modulates STAT1 activity, but not others, is probably specific to cell type (RTECs) or stimilus (IRI), while the underlying mechanisms are worth investigating further. Overall, our findings provide novel insights into the regulatory mechanism of RTEC pyroptosis during AKI by the DUSP2-STAT1 axis.

In conclusion, this study demonstrates a hitherto unrecognized role of the DUSP2-STAT1 axis in regulating RTEC pyroptosis during AKI. The findings of our study not only provide novel insights into the pathogenesis and treatment of AKI, but also advance our understanding of the intrinsic regulation of pyroptotic cell death. Targeting the DUSP2-STAT1-pyroptosis axis may be an attractive therapeutic strategy for AKI.

## Supplementary Material

Supplementary figures and tables.

## Abbreviations

AKI: Acute kidney injury
RTEC: Renal tubular epithelial cell
DUSP2: Dual-specificity phosphatase 2
CoIP: Coimmunoprecipitation
ChIP: chromatinimmunopercitation
PAC-1: Phosphatase of activated cells 1
MAPKs: Mitogen-activated protein kinases
H/R: Hypoxia/reoxygenation
TEM: Transmission electron microscope
SEM: Scanning electron microscopy
AAV: Adeno-associated virus
LDH: Lactate dehydrogenase
