# Supplementary material for: DUSP2-mediated inhibition of tubular epithelial cell pyroptosis confers nephroprotection in acute kidney injury
Source: Theranostics. 2022 Jul 4;12(11):5069–85. doi: 10.7150/thno.72291 (PMC9274747; doi:10.7150/thno.72291)
Supplement: Supplementary file 1 — Supplementary figures and tables. [file thnov12p5069s1.pdf]

Supplementary Figures and Tables

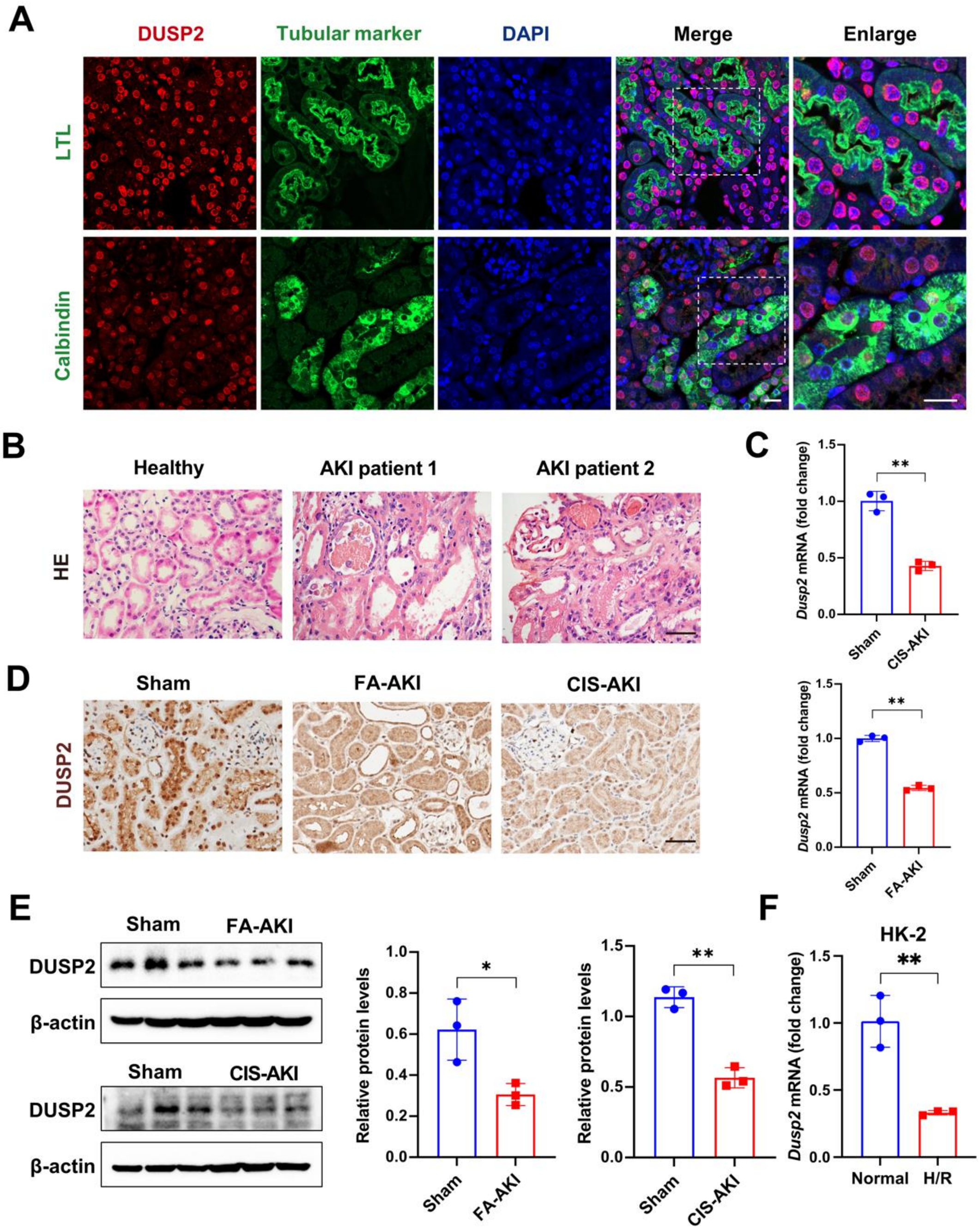

Figure S1: Loss-of-DUSP2 in RTECs is common in AKI.

(A) Representative immunofluorescent images of DUSP2, lotus tetragonolobus lectin (LTL, proximal tubular marker), and Calbindin (distal tubular marker) in the kidneys from healthy mice. Scale bars, 20 μm. (B) Representative H&E images of the kidneys

from healthy controls and patients with AKI. The paracarcinoma tissues from patients without nephropathy were used as healthy control. Scale bar, 50 μm. (C) Relative mRNA expression levels of *Dusp2* in the renal cortexes from folic acid-induced (FA-AKI) and cis-platinum induced AKI (CIS-AKI) mice (n = 3). (D-E) The expressions of DUSP2 in the kidneys from FA-AKI and CIS-AKI mice (n = 3). Scale bar, 50 μm. (F) Relative mRNA expression levels of *Dusp2* in HK-2 cells with or without H/R injury. Data are presented as mean±SD. *$p < 0.05$; **$p < 0.01$; compared with the sham or normal groups.

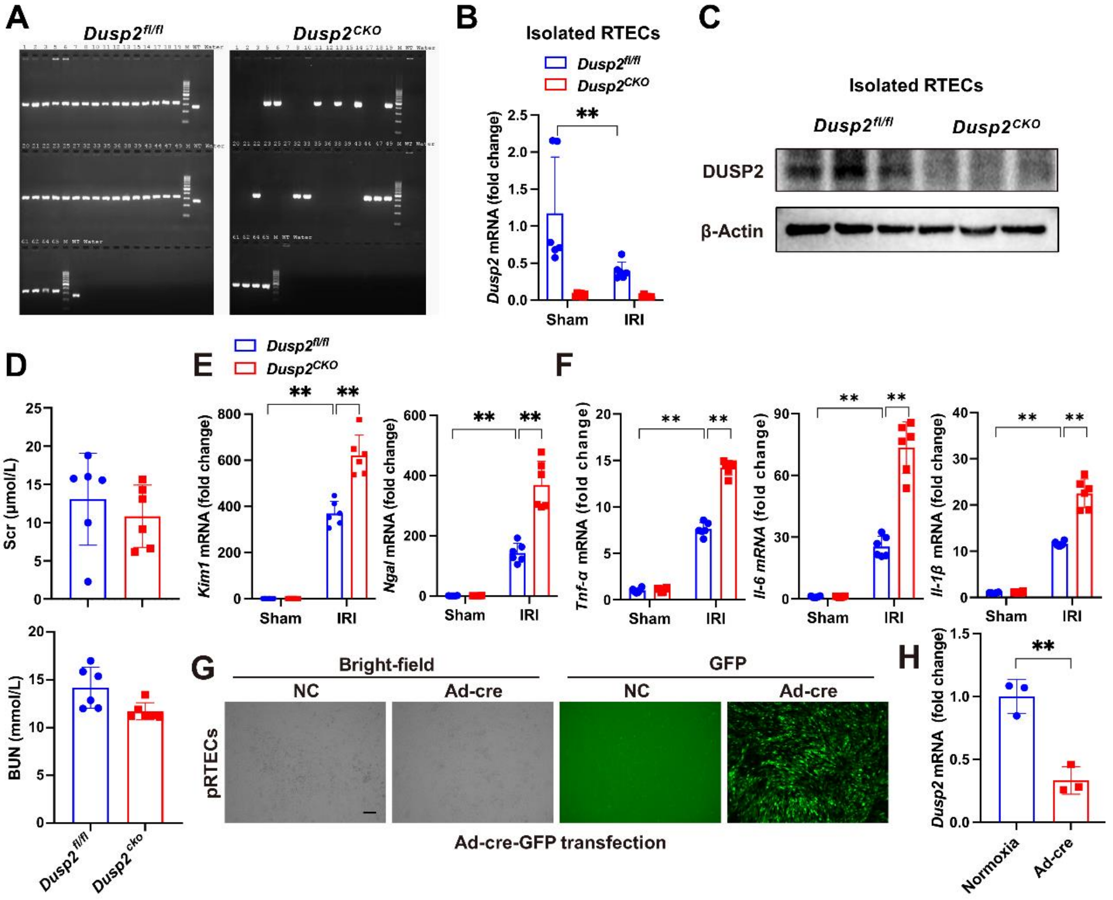

Figure S2: RTEC-specific deletion of DUSP2 aggravates IRI-induced renal inflammation. (A) The genetic identification of *Dusp2*$^{fl/fl}$ and *Dusp2*^{CKO} mice. (B-C) Relative mRNA (B) and protein (C) expression levels of DUSP2 in isolated RTECs from *Dusp2*$^{fl/fl}$ and *Dusp2*^{CKO} mice (n = 6). (D) The measurements of Scr and BUN of *Dusp2*$^{fl/fl}$ and *Dusp2*^{CKO} mice (n = 6). (E-F) Relative mRNA expression levels of renal tubular injury markers *Kim1* and *Ngal* (E) as well as the inflammatory factors *Tnf-α, Il-6*, and *Il-1β* (F) in the renal cortexes from *Dusp2*$^{fl/fl}$ and *Dusp2*^{CKO} mice with or without IRI (n = 6). (G) Representative immunofluorescent images of the Ad-cre-GFP. Scale bar, 50 μm. (H) The mRNA expression of *Dusp2* in isolated RTECs from *Dusp2*$^{fl/fl}$ mice treated with Ad-cre-GFP (n = 3). Data are presented as mean±SD. *$p < 0.05$; **$p < 0.01$; compared with the indicated group.

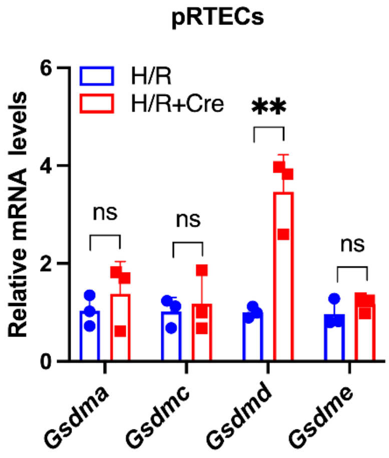

Figure S3. The mRNA expression levels of gasdermins in pRTECs post-H/R. Relative mRNA expression levels of gasdermins in pRTECs treated with or without Ad-cre-GFP before H/R injury. Data are presented as mean±SD. ***p* < 0.01; compared with the H/R group.

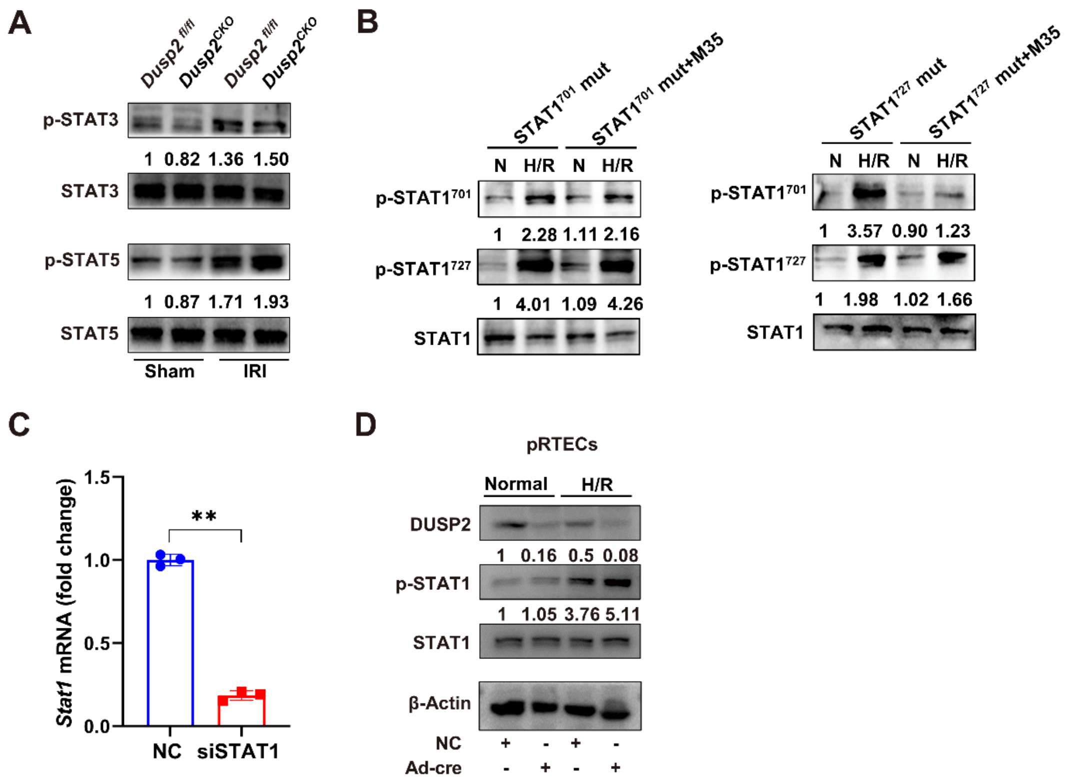

Figure S4: DUSP2 deactivates STAT1 *in vitro*. (A) The protein expression levels of STAT3, p-STAT3, STAT5, and p-STAT5, in the renal cortexes from *Dusp2*$^{fl/fl}$ and *Dusp2*^{CKO} mice with or without IRI. (B) The protein expression levels of p-STAT1 and STAT1 in STAT1 Tyr^{701} mutated or Ser^{727} mutated HK-2 cells with or without DUSP2 overexpression prior to normoxia or H/R treatments. (C) Relative mRNA expression levels of *Stat1* in HK-2 cells with or without STAT1 RNAi. (D) The protein expression levels of DUSP2, p-STAT1, and STAT1, in DUSP2-deficient pRTECs with or without H/R injury. Data are presented as mean±SD. $*p < 0.05$; $**p < 0.01$; compared with the indicated group.

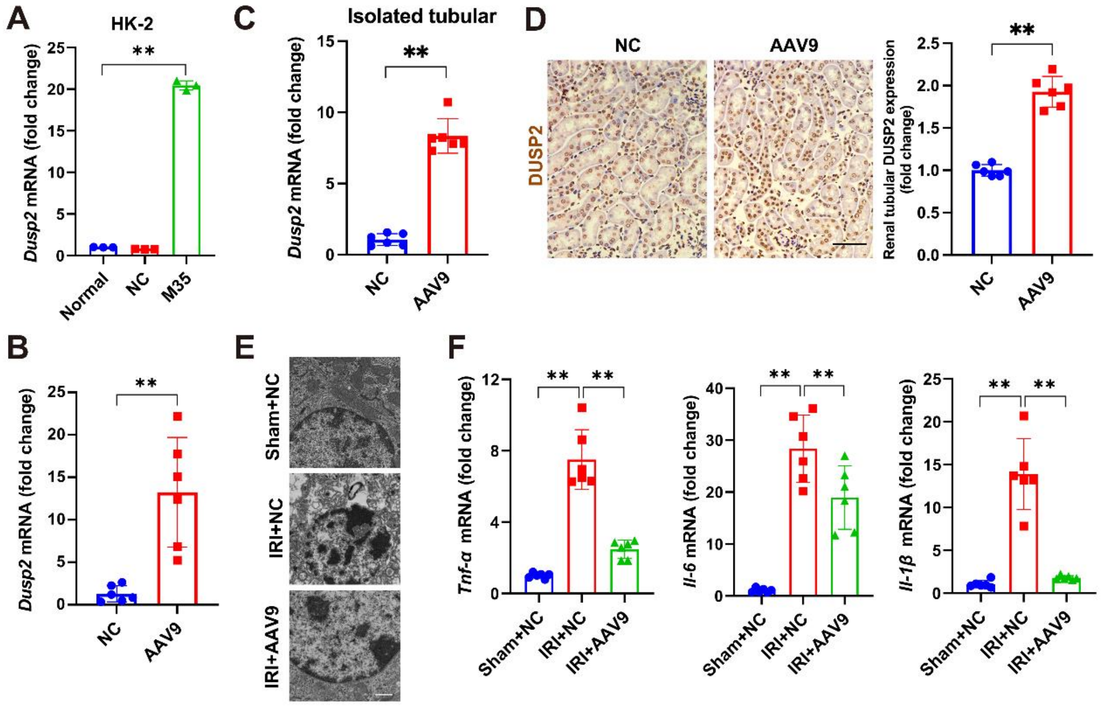

Figure S5: DUSP2 overexpression in RTECs protects against AKI. (A) Relative mRNA expression levels of *Dusp2* in HK-2 cells with or without DUSP2 overexpression. (B-C) Relative mRNA expression levels of *Dusp2* in the renal cortexes (B) or the isolated tubules (C) of mice injected with or without AAV-*Dusp2* (n = 6). (D) Representative IHC staining of DUSP2 in the kidneys from mice injected with or without AAV-*Dusp2* (n = 6). Scale bar, 50 μm. (E) Representative TEM images of pyroptosis in the kidneys from mice injected with or without AAV-*Dusp2* before being subjected to IRI. Scale bar, 1 μm. (F) Relative mRNA expression levels of the inflammatory factors *Tnf-α, Il-6*, and *Il-1β* (n = 6). Data are presented as mean±SD. **p* < 0.05; ***p* < 0.01; compared with the indicated group.

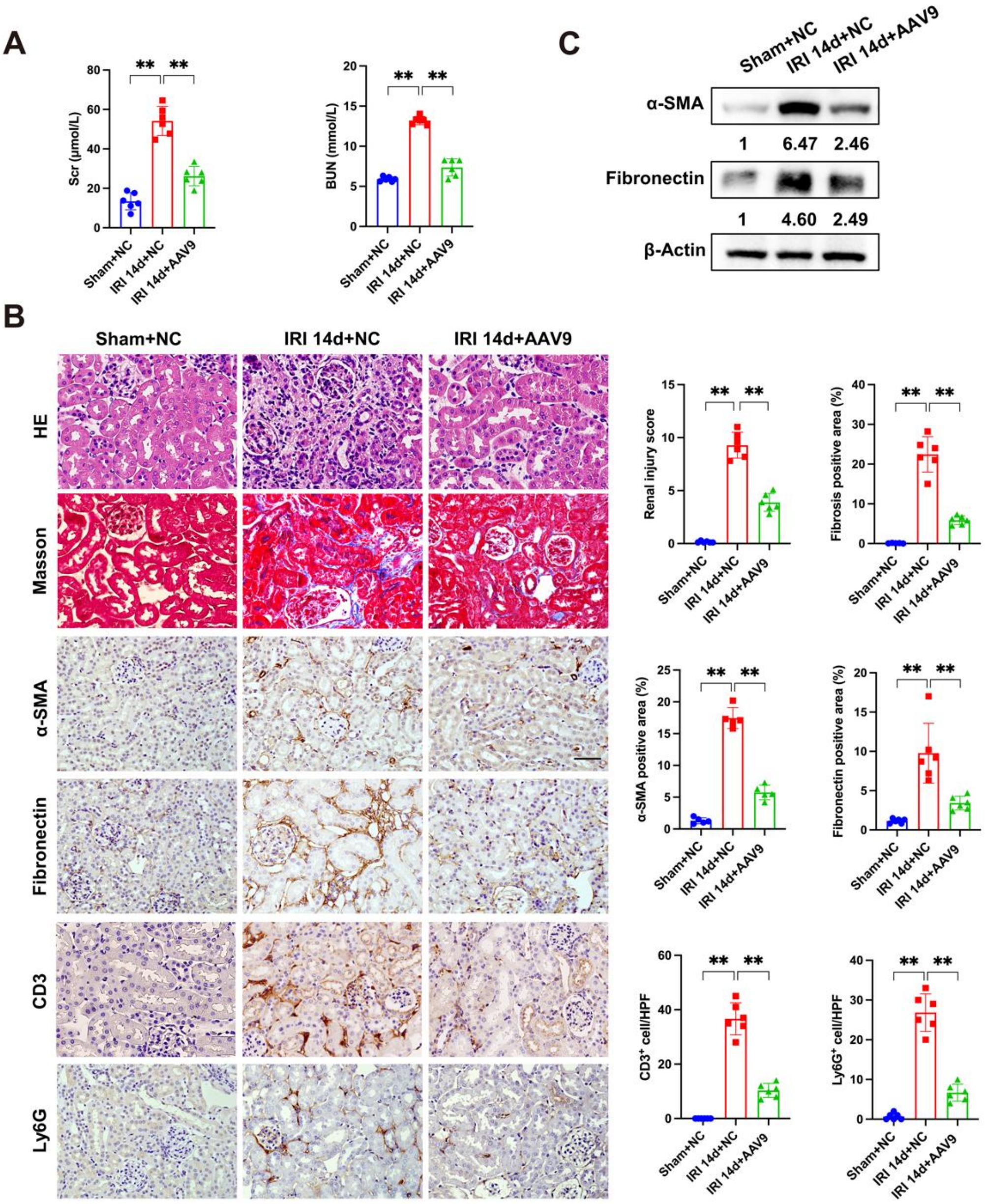

Figure S6. Overexpression of DUSP2 in RTECs inhibits renal fibrosis.

(A) The measurements of Scr and BUN in mice subjected to IRI for 14 days after NC or AAV-*Dusp2* administrations (n = 6). (B) Representative images of H&E, Masson, as well as IHC staining of α-SMA, Fibronectin, and inflammatory markers (CD3 and Ly6G). Scale bars: 50 μm. (C) Western blot analysis of α-SMA and Fibronectin. Data are presented as mean±SD. *$p < 0.05$; **$p < 0.01$; compared with the indicated group.

Table S1. The basic characteristic of the included AKI patients

Patients No.	Age (years)	Sex	Diagnosis	BUN (mmol/L)	eGFR (ml/min per 1.73 m^2)	serum creatine (mg/dL)
1	62	Male	AKI	38.27	5	9.29
2	25	Male	AKI	6.83	18	4.31
3	31	Male	AKI	5.33	74	1.28
4	55	Male	AKI	27.84	6	8.45
5	24	Male	AKI	25.13	28	3.01
6	17	Female	AKI	21.09	7.7	7.13
7	63	Male	AKI	14.32	4	10.95
8	70	Female	AKI	26.53	5	8.01
9	67	Male	AKI	16.59	25	2.56
10	21	Male	AKI	10.55	26	3.21
11	69	Male	AKI	18.17	6	7.96
12	32	Male	AKI	12.4	13	5.50
13	24	Male	AKI	14.48	5.85	10.88
14	28	Male	AKI	9.46	19.3	3.96
15	72	Female	AKI	9.84	6.9	5.68
15	70	Male	AKI	18.87	4.4	10.45
16	55	Male	AKI	28.44	5.12	10.16
18	51	Male	AKI	22.52	8.44	6.87

Table S2. siRNA target sequences

siRNA	Organisms	Sequences (5' to 3')
siSTAT1	Mus musculus	Sense: GGAUAACUUCCAAGAAGAUTT
		Antisense: AUCUUCUUGGAAGUUAUCCTT
siSTAT1	Homo sapiens	Sense: CGAACAUGACCCUAUCACATT
		Antisense: UGUGAUAGGGUCAUGUUCGTT
siGSDMD	Mus musculus	Sense: GAUGUCGUCGAUGGGAACAUU
		Antisense: AAUGUUCCCAUCGACGACAUC

Table S3. The antibodies used in the current study				
**NO.**	**Antibodies**	**Experiment**	**Source**	**Identifier**
1	Anti-DUSP2	Immunohistochemistry, immunofluorescence staining	BIOSS antibiotics	bs-7609R
2	Anti-DUSP2	Western blot	Cohesion Biosciences	#CQA4311
3	Anti-DUSP2	Flow cytometry	Invitrogen	PA5-26093
4	Anti-Phospho-Stat1	Western blot, immunofluorescence staining	Cell Signaling Technology	#7649
5	Anti-Stat1	Western blot, immunofluorescence staining	Cell Signaling Technology	#14994
6	Anti-Phospho-Stat3	Western blot	Cell Signaling Technology	#9145
7	Anti-Stat3	Western blot	Cell Signaling Technology	#9139
8	Anti-Phospho-Stat5	Western blot	Cell Signaling Technology	# 4322
9	Anti-Stat5	Western blot	Cell Signaling Technology	# 94205
10	Anti-GSDMD	Western blot	Abcam	ab219800
11	Anti-GSDMD	Western blot	Abcam	ab210070
12	Anti-GSDMD-N	Immunohistochemistry, immunofluorescence staining	Novus Biologicals	NBP2-80427
13	Anti-β-Actin	Western blot	Cell Signaling Technology	#3700
14	Anti-CD3	Immunofluorescence staining	Santa Cruz Biotechnology	sc-20047
15	Anti-Ly-6G	Immunofluorescence staining	Santa Cruz Biotechnology	sc-53515
16	Anti-F4/80	Immunofluorescence staining	Abcam	ab6640
17	Anti-IL-1 beta	Flow cytometry	Novus Biologicals	NB600-633
18	Anti-Lotus Tetragonolobus Lectin (LTL)	Immunofluorescence staining	Vector Laboratories	FL-1321
19	Anti-Calbindin	Immunofluorescence staining	BIOSS antibiotics	bs-3758R

Table S4. Primer sequences for qPCR			
**Gene**	**Organisms**	**Forward (5' to 3')**	**Reverse (5' to 3')**
Dusp2	*Homo sapiens*	CTTCCTGCGAGGAGGCTTCG	CTGCAGGTCTGACGAGTGAC
Dusp2	*Mus musculus*	TGTGGAAATCTTGCCCTACCT	CCCACTATTCTTCACCGAGTCTA
Actb	*Mus musculus*	AACAGTCCGCCTAGAAGCAC	CGTTGACATCCGTAAAGACC
Actb	*Homo sapiens*	CATGTACGTTGCTATCCAGGC	CTCCTTAATGTCACGCACGAT
Kim-1	*Mus musculus*	*ACATATCGTGGAATCACAACGAC*	ACTGCTCTTCTGATAGGTGACA
Ngal	*Mus musculus*	GCAGGTGGTACGTTGTGGG	CTCTTGTAGCTCATAGATGGTGC
Il-6	*Mus musculus*	GCCTTCTTGGGACTGATGCT	GCCATTGCACAACTCTTTTCTCA
Tnf-α	*Mus musculus*	ACTCAGAAACACAAGATGCT	CAGAACTCAGGAATGGACAT
Il-1β	*Mus musculus*	TTCAGGCAGGCAGTATCACTC	CCAGCAGGTTATCATCATCA
Stat1	*Mus musculus*	TCACAGTGGTTCGAGCTTCAG	GCAAACGAGACATCATAGGCA
Gsdma	*Mus musculus*	AGGTAGGTGCACGGCTTACA	AGGAGATGGCTGAGGGAAGT
Gsdmc	*Mus musculus*	ACTGAAGGCTGACCTGGAT	TAAATGTGGGCAACTGAT
Gsdmd	*Mus musculus*	GAAAGCGAAGCTCCCGGAT	TCCGAAGCTGTTGCAGGATT
Gsdme	*Mus musculus*	TGAGGAAGCAGGAGGTGG	CATTGGTGTCCGTGGTGA
Casp3	*Mus musculus*	ATGGAGAACAACAAAACCTCAGT	TTGCTCCCATGTATGGTCTTTAC
Mlkl	*Mus musculus*	TTGACTTTAGGCGGGAACCG	CCAGGGCAGCAGTAATGTCA
Ripk3	*Mus musculus*	GCCTTCCTCTCAGTCCACAC	CTCACCAGAGGAACCGCATA
Gpx4	*Mus musculus*	CGCCAAAGTCCTAGGAAACG	TATCGGGCATGCAGATCGAC
Slc7a11	*Mus musculus*	AATACGGAGCCTTCCACGAG	ACTGTTCGGTCGTGACTTCC
Casp1	*Mus musculus*	ACTGCTATGGACAAGGCACG	GCAAGACGTGTACGAGTGGT

Table S5. Primer sequences for qPCR for ChIP			
**Gene**	**Organisms**	**Forward (5' to 3')**	**Reverse (5' to 3')**
P1	*Homo sapiens*	AGGGAGAAAGTGACAGTGGGAGA	CAGCCTGGGTGACAGAGCAA
P2	*Homo sapiens*	GGTCAGGCATTGCCATCAGG	CACTTTGCTAGAAGAAGCCGTCA
P3	*Homo sapiens*	AACCTCTGCTTCCCAAGTTCAA	TGGCTCATGCCTGTAATCCC
P4	*Homo sapiens*	CTGGCAGTGACGGCTTCTTC	CAGGTCTGAGGTGGGCTTGA
P5	*Homo sapiens*	GGCTCTTCTGCCACCTGCCTCT	CTCCAGGGCTTTGGGCGTCT
P6	*Homo sapiens*	AGAAGCCAGCGAGGAGTGAG	CCAGACCGCGACCTGGACAA
P7	*Homo sapiens*	GTGAGTCCTCGTGCCCTTCC	CCTGGTTCTAGGAGCCAAGACAA
N1	*Homo sapiens*	ATGTGGTGCTGAGGCAGAGC	GAGGCCCAGAGCTAGAGGCT
N2	*Homo sapiens*	TCACAACCTTGGGGCATCAG	TCCTTCCTGCAAGCTGGTTC
N3	*Homo sapiens*	GGACAAGGGGTGGTGTGAAC	AAAGGTGGACTCGGGGACTC
